# Inhibitory and Agonistic Autoantibodies Directed Against the β_2_-Adrenergic Receptor in Pseudoexfoliation Syndrome and Glaucoma

**DOI:** 10.3389/fnins.2021.676579

**Published:** 2021-08-06

**Authors:** Bettina Hohberger, Ursula Schlötzer-Schrehard, Christian Mardin, Robert Lämmer, Luis Munoz, Rudolf Kunze, Martin Herrmann, Gerd Wallukat

**Affiliations:** ^1^Department of Ophthalmology, Friedrich-Alexander-University of Erlangen-Nuremberg, Erlangen, Germany; ^2^Department of Internal Medicine III, Institute of Clinical Immunology and Rheumatology, University of Erlangen-Nuremberg, Erlangen, Germany; ^3^Science Office, Berlin-Buch, Campus Max Delbrück Center for Molecular Medicine, Berlin, Germany; ^4^Berlin Cures GmbH, Berlin, Germany

**Keywords:** autoantibodies, glaucoma, pseudoexfoliation, β_2_-adrenergic receptor, imbalanced autonomic theory

## Abstract

Pseudoexfoliation syndrome (PEXS) and glaucoma (PEXG) are assumed to be caused by a generalized elastosis leading to the accumulation of PEX material in ocular as well as in extraocular tissues. The exact pathophysiology of PEXS is still elusive. PEXG, the most common type of secondary open-angle glaucoma (OAG), is characterized by large peaks of intraocular pressure (IOP) with a progressive loss of the visual field. Agonistic autoantibodies (agAAbs) against the β_2_-adrenergic receptor (AR) have been shown to be present in sera of patients with primary and secondary OAG and ocular hypertension and are seemingly linked to IOP. In the present study, we investigated the autoantibodies directed against the β_2_-AR in sera of patients with PEXS and PEXG. We recruited 15, 10, and 15 patients with PEXG, PEXS, and primary OAG, respectively. Ten healthy individuals served as controls. All patients underwent standard ophthalmological examination with Octopus G1 perimetry. agAAbs prepared from serum samples were analyzed in a rat cardiomyocyte–based bioassay for the presence of agAAbs. We identified the interacting loop of the β_2_-AR and the immunoglobulin G (IgG) subclasses using synthetic peptides corresponding to the extracellular loops of the receptors and enzyme-linked immunosorbent assay, respectively. None of the controls were β_2_-agAAb–positive (0.2 ± 0.5 U). No β_2_-agAAbs (0.2 ± 0.4 U), but inhibitory β_2_-AAbs were observed in 80% of the patients that partially blocked the drug-induced β_2_-adrenergic stimulation; 5.8 ± 1.7 U vs. 11.1 ± 0.9 U for clenbuterol in the absence and the presence of sera from patients with PEXS, respectively. Epitope analyses identified the third extracellular loop of the β_2_-AR as the target of the inhibitory β_2_-AAbs, being of IgG3 subtype in PEXS patients. In contrast, patients with PEXG showed β_2_-agAAbs (5.6 ± 0.9 U), but no inhibitory ones. The β_2_-agAAbs levels of patients with PEXG and primary OAG patients (3.9 ± 2.8 U; *p* > 0.05) were at a similar level. In two cases of PEXG, the β_2_-agAAbs exert synergistic effects with clenbuterol. The activity increased from 11.5 ± 0.3 (clenbuterol only) to 16.3 ± 0.9 U. As autoimmune mechanisms were reportedly involved in the pathogenesis of glaucoma, agonistic and inhibitory β_2_-AAbs seem to be a part of this multifactorial interplay.

## Introduction

Pseudoexfoliation syndrome (PEXS) is an age-related disorder affecting the extracellular matrix of the whole body (Elhawy et al., 2012; Scharfenberg and Schlotzer-Schrehardt, 2012). It is characterized by the pathological accumulation of extracellular material (i.e., PEX material) in intraocular and extraocular tissues (Ritch and Schlotzer-Schrehardt, 2001; Elhawy et al., 2012). PEX material consists of fibrillar aggregates containing elastic fiber components such as elastin, fibrillin-1, fibulin, microfibril-associated glycoprotein-1 (MAGP-1), latent transforming growth factor β (LTBP)1/2, and cross-linking enzymes such as lysyl oxidase-like 1 (LOXL1) (Schlotzer-Schrehardt et al., 2000; Krumbiegel et al., 2009; Lee et al., 2009; Zenkel and Schlotzer-Schrehardt, 2014; Founti et al., 2015), which can accumulate in the trabecular meshwork, the main outflow pathway of the eye, and may reduce the outflow of aqueous humor and increased intraocular pressure (IOP) (Johnson and Brubaker, 1982). Consequently, PEXS is strongly associated with the development of PEX glaucoma (PEXG) (Schlotzer-Schrehardt et al., 2002; Anastasopoulos et al., 2015). The latter is the most common type of secondary open-angle glaucoma (SOAG) (Ritch, 2001). The pathophysiology of PEXS and PEXG is still elusive (Anastasopoulos et al., 2015; Schlotzer-Schrehardt and Khor, 2021). It is widely assumed that a generalized elastosis, induced by stress, is the principal process in the pathophysiology of PEXS (Schlotzer-Schrehardt et al., 2002; Anastasopoulos et al., 2015; Schlotzer-Schrehardt and Khor, 2021). Transforming growth factor β1 (TGF-β1), being increased in aqueous humor of PEXG and PEXS, and oxidative stress were considered to be the main contributors to the disease (Schlotzer-Schrehardt et al., 2001; Schlotzer-Schrehardt, 2010). PEX is reportedly associated with an increased risk of vascular diseases like myocardial infarction, coronary artery disease, aortic aneurysms, stroke, and cerebrovascular disease (Mitchell et al., 1997; Praveen et al., 2011; Yokusoglu, 2012; Gonen et al., 2013; Andrikopoulos et al., 2014; Siordia et al., 2016). Again the underlying mechanisms are not fully understood (Mitchell et al., 1997; Siordia et al., 2016). As with other glaucomas, the main target in treating PEXG is the increased IOP (Schlotzer-Schrehardt et al., 2002; Anastasopoulos et al., 2015). All conservative, laser, or surgical strategies aim to lower IOP up to an individual target level in order to win sighted lifetime (Lusthaus and Goldberg, 2019; Cvenkel and Kolko, 2020). Reduction of IOP by 1 mm Hg is known to prevent the visual field loss of patients by 10% (Leske et al., 2003) and should be made strictly forward (Musch et al., 2011). A main antiglaucomatous medication is timolol, a blocker of the β_2_-adrenergic receptor (AR) (Katz et al., 1976; Barnes and Moshirfar, 2021), that reduces the formation of aqueous humor by up to 50% (Coakes and Brubaker, 1978; Yablonski et al., 1978; Dailey et al., 1982; Topper and Brubaker, 1985; Mccannel et al., 1992; Wayman et al., 1997; Larsson, 2001). As β_1_-AR blockers (e.g., betaxolol) reportedly lower IOP less effectively than β_2_-AR blockers, the role of the latter in regulating IOP appears to be predominant (Gaul et al., 1989). β_2_-ARs are expressed on cells of the ciliary body and the trabecular meshwork, the tissues involved in aqueous humor production and outflow (Wax and Molinoff, 1987; Crider and Sharif, 2002). Cells of the optic nerve and retinal pericytes are further tissues that express β_2_-AR (Mantyh et al., 1995; Feher et al., 2005). agAAbs against β_2_-AR (β_2_-agAAbs) have been detected in the sera of patients with primary open-angle glaucoma (POAG) and SOAG, as well as ocular hypertension (OHT) (Junemann et al., 2018; Hohberger et al., 2019a). These agAAbs were mainly of the immunoglobulin G3 (IgG3) subtype and were directed against the second extracellular loop of the β_2_-AR (peptide 181–192) (Junemann et al., 2018). The immunological targeting of the β_2_-AR is the basis for the hypothesis of an involvement of β_2_-agAAbs in dysregulation of IOP (Zimmerman, 1977). The IOP is the result of production (ciliary body) and outflow (trabecular meshwork, uveosclera) of the aqueous humor (Johnstone et al., 2020; Kaufman, 2020; Sunderland and Sapra, 2021). Clinical data of patients with POAG, showing a reduced IOP after removal of all circulating IgG antibodies by immunoadsorption, support this hypothesis (Junemann et al., 2018). These findings indicated a potential role for autoimmune processes in the dysregulation of IOP. As patients with PEXG typically show an increased IOP with large fluctuations (Heijl et al., 2009), we hypothesize a role of the β_2_-agAAbs in the development of PEXG. Various types of agAAbs, directed against the β_2_-AR, were described in literature (Wallukat and Wollenberger, 1991; Wallukat, 2002; Junemann et al., 2018): agonistic and inhibitory AAbs activate and block the β_2_-AR, respectively. It was the aim of this study to analyze serum samples from patients with PEXS and PEXG for agAAbs against β_2_-AR.

## Materials and Methods

### Patients and Controls

Fifty subjects were recruited from the Department of Ophthalmology, University of Erlangen-Nuremberg: patients [*n* = 40; PEXG (*n* = 15), PEX (*n* = 10), POAG (*n* = 15)] and controls (*n* = 10). All patients underwent a complete ophthalmological examination including slit-lamp biomicroscopy, funduscopy, and measurement of IOP by Goldmann applanation tonometry (Haag Streit, Koeniz, Switzerland). In addition, we performed standard white-on-white full-field perimetry (Octopus 500, G1 protocol; Interzeag, Schlieren, Switzerland).

#### PEX Glaucoma

Diagnosis of PEXG was based on an open anterior chamber angle, the presence of PEX material on anterior segment structures, repeated elevated IOP > 21 mm Hg, glaucomatous alterations of the optic disc according to Jonas’ classification (Jonas et al., 1988), and perimetric field loss, confirmed at least once.

#### PEX Syndrome

Patients with PEXS showed PEX material deposits in the anterior chamber, but without any signs of glaucoma (Scharfenberg and Schlotzer-Schrehardt, 2012; Hohberger et al., 2019b).

#### POAG

Diagnosis of POAG was based on the glaucoma criteria for PEXG, yet without any signs of PEX material on ocular structures (Jonas et al., 1988; Barnes and Moshirfar, 2021).

#### Controls

Tonometry, slit-lamp inspection, funduscopy, and papillometry were normal in control subjects. No signs of PEX or glaucoma were observed, yet the presence of cataract was allowed.

Blood samples were collected according to clinical all-day life procedures and centrifuged, and sera were stored at −80°C previous to analysis. All individuals declared informed consent. The study was performed according to the tenets of the Declaration of Helsinki and approved by the Ethic Committee of the University of Erlangen and the Max-Delbrück-Centrum for Molecular Medicine, Berlin-Buch (Y 9004/19, 02.11.2020).

### Cardiomyocyte Bioassay

The cardiomyocyte bioassay was used as described previously (Junemann et al., 2018). Cell culture of cardiac myocytes (heart ventricle of 1–3-day-old Sprague–Dawley rats) was digested with a 0.25% solution of crude porcine trypsin (Serva, Germany). The cells were dispersed and suspended in SM20-I medium (Biochrom, Germany), glutamine (Serva, Germany), which contained streptomycin (HEFA Pharma, Germany), penicillin (Heyl, Germany), hydrocortisone (Merck, Germany), 10% heat-inactivated neonatal calf serum (Gibco, Germany), and fluorodeoxyuridine (Serva, Germany). The cells were seeded with a field density of 160,000 cells/cm^2^. After 24 h, the culture medium was replaced, respectively. Before stimulation, the cardiomyocytes were cultured for 3–4 days (37°C). Two hours before the experiment, the medium was renewed. After incubation with the immunoglobulin fractions of the sera of each proband (1:40, for 60 min), the beating rates (BRs) of the cardiomyocytes were counted for 7–10 spontaneously beating cells for 15 s at a heated stage inverted microscope (37°C). One unit reflects 4 beats/min (i.e., adrenergic activity). Samples of less than 2 units of adrenergic activity were considered as normal. Differentiation between agonistic and inhibitory β_2_-AAbs was done after additional incubation with 3 μM clenbuterol; the latter one partially blocked the clenbuterol effect. Application of ICI-118,551 (2 μL ICI to 2 mL medium in order to avoid dilution) was done for identification of the receptor type (β_2_-receptor).

### Preparation of the Serum Immunoglobulins

The IgGs were prepared from sera of patients by direct ammonium sulfate precipitation (final concentration of 40%) overnight at 4°C. Afterward, the precipitates were centrifuged (4,000 × *g*, 30 min). The pellets were dissolved in dialysis buffer (154 mmol/L NaCl, 10 mmol/L Na_2_HPO_4_/NaH_2_PO_4_, pH 7.2). Precipitating, washing, and dissolving were repeated twice. The samples were dialyzed against phosphate-buffered saline (4°C, 3 days) prior to application to the cardiomyocyte bioassay. The samples were stored at –20°C.

### Characterization of the Target Loop of Inhibitory AAbs Against β_2_-AR

Characterization of the target loop was done as described previously (Junemann et al., 2018). Synthetic peptides corresponding to the extracellular loops I, II, and III of the human β_2_-AR were used to identify the molecular domain of the inhibitory β_2_-AAbs:

1. loop I (HILMKMWTFGNFWCEFWT).
2. loop II (HWYRATHQEAINCYANETCCDFFTNQ).
3. loop III (VIQDNLIRKEV).

After shaking 0.5 μg peptide in 50 μL IgG fraction, they were incubated for 1 h at room temperature before adding them to the bioassay (final IgG dilution of 1:40).

### Analysis of IgG Subclass of the Inhibitory AAbs Against β_2_-AR

IgG subclass analysis of the inhibitory AAbs was done as described previously (Junemann et al., 2018). IgGs (50 μL) from selected patients were treated with murine monoclonal anti-human IgG1, IgG2, IgG3, and IgG4 antibodies (3 μL, SEROTEC, Germany). After incubation (1 h, room temperature), each sample was treated with 3 μL of a polyclonal anti-mouse Fc antibodies (1 h) in order to increase the immune complex. Centrifugation of the samples (at 10,000 *g*) was done, and the supernatants were added to the cardiomyocyte bioassay (see above; dilution of 1:40).

### Statistical Analysis

Statistical calculations were done with Microsoft Excel 2016 and the GraphPad Prism Software version 8.3. Data are shown as means and standard errors of the mean for each group. The Kolmogorov–Smirnov test was run prior to analysis for testing the normal distribution. According to this, the one-way analysis of variance (normally distributed data) and the Kruskal–Wallis test (non–normally distributed data) with a post-test for multiple comparisons were applied, respectively. When only two groups were compared, the Mann–Whitney test or the Student’s *t*-test was applied, accordingly. *p* < 0.05 was considered significant. A list of all abbreviations can be seen in Table 1.

**TABLE 1:** List of abbreviations.

PEXS	Pseudoexfoliation syndrome
PEXG	Pseudoexfoliation glaucoma
OAG	Open-angle glaucoma
POAG	Primary open-angle glaucoma
SOAG	Secondary open-angle glaucoma
OHT	Ocular hypertension
IOP	Intraocular pressure
agAAbs	Autoantibody
β_2_-AAbs	Autoantibody against the adrenergic β_2_-adrenoreceptor
β_2_-agAAbs	Agonistic autoantibody against the adrenergic β_2_-adrenoreceptor
β_2_-AR	Adrenergic β_2_-adrenoreceptor
CB	Clenbuterol
IgG	Immunoglobulin G
MAGP-1	Microfibril-associated glycoprotein-1
LTBP	Latent transforming growth factor β
LOXL1	Lysyl oxidase-like 1
Transforming growth factor	Transforming growth factor
IL	Interleukin
BR	beating rate

## Results

We analyzed the sera of patients with PEXS and PEXG and controls for the presence of agonistic and inhibitory AAbs directed against the β_2_-AR. None of the control samples showed a β_2_-agAAb seropositivity (neither agonistic nor inhibitory) of 0.2 ± 0.5 U in the cardiomyocyte bioassay. The cutoff was set at mean ± 3 standard deviation of the controls (1.66 U).

### Sera of Patients With PEXS Contain Inhibitory but No Agonistic β_2_-AAbs

**FIGURE 1:**
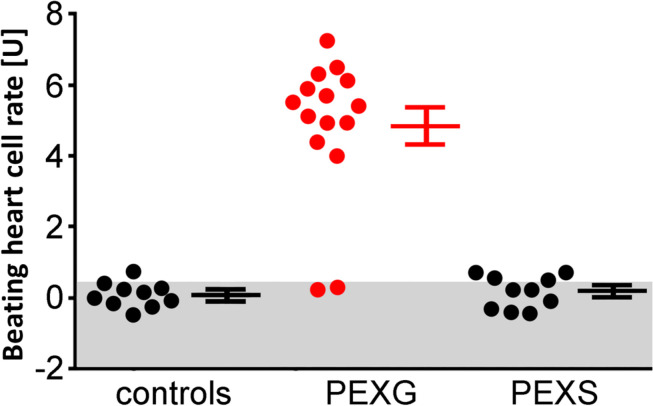
Adrenergic activity of β_2_-AAbs in sera of patients with PEX syndrome (PEXS), PEX glaucoma (PEXG), and controls. Beating heart cell rates were shown for each group [absolute data, mean ± standard deviation (SD)]: controls, patients with PEXG and PEXS. One unit (U) reflects 4 beats/min. The values represent the adrenergic activity of β_2_-Abs. Samples less than 2 units of adrenergic activity were considered as normal. Increased beating heart cell rate was to be observed for patients with PEXG (red) when compared to patients with PEXS and controls as measured by rat cardiomyocyte beating heart cell rates.

**FIGURE 2:**
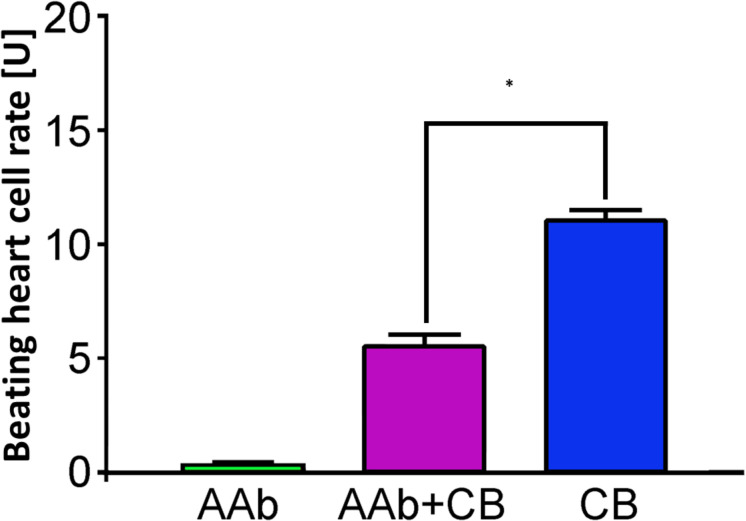
β_2_-AAbs in patients with PEXS are of inhibitory nature. Beating heart cell rates after single application of sera of patients with PEXS without (green) and with additional clenbuterol (CB, pink); reference: single incubation with CB (blue); 1 unit (U) reflects 4 beats/min; mean ± standard deviation (SD). β_2_-AAbs inhibit the increased beating heart cell rate, due to the effect of clenbuterol, and are thus of inhibitory nature. *p < 0.001, with a post-test for multiple comparisons.

None of the 10 patients with PEXS showed β_2_-agAAbs (mean = 0.2 ± 0.1 U) (Figure 1). Clenbuterol alone increased the BR value of the cardiomyocytes by 11.1 ± 0.5 U. After coincubation of the IgG with 3 μM clenbuterol (mean), a decreased BR value was observed in 8 (80%) of 10 PEXS patients (5.8 ± 0.6 U, *p* < 0.001). This decrease was attributed to the inhibitory β_2_-AAbs that partially blocked the clenbuterol effect (*p* < 0.001; Figure 2).

### The Inhibitory β_2_-AAbs Recognize the Third Extracellular Loop of the β_2_-AR

**FIGURE 3:**
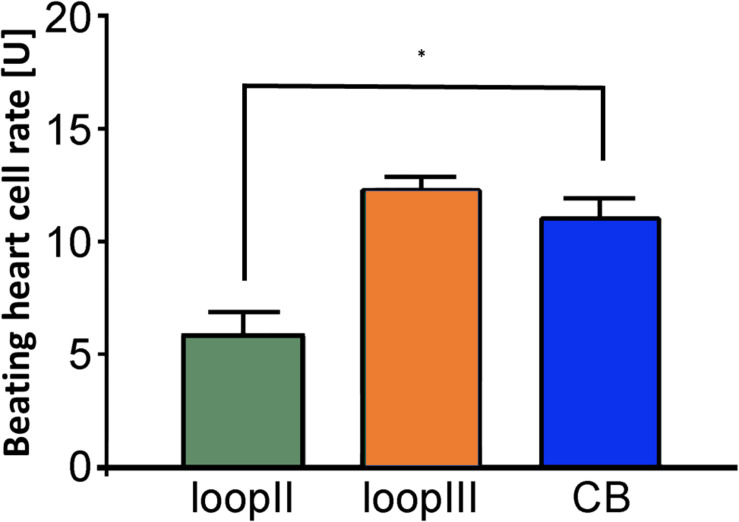
Epitope mapping of the inhibitory anti–β_2_-AAb in patients with PEX syndrome revealed that inhibitory anti–β_2_-AAb targets extracellular loop III. Peptides of the β_2_-adrenergic receptor extracellular loops II and III were used for epitope mapping of the inhibitory anti–β_2_-AAb in patients with PEX syndrome in the presence of clenbuterol (Cb): peptides against the third loop did block the binding site of the inhibitory anti–β2-AAb. Consequently, additional application of Cb did show an increase in the beating rate (orange); peptides against the second loop did not block the binding site of the inhibitory anti–β2-AAb. Consequently, the inhibitory nature of the inhibitory anti–β2-AAb reduced significantly the Cb effect (^∗^*p* = 0.001, green); application of clenbuterol alone (control, blue); 1 unit (U) reflects 4 beats/min; mean ± standard deviation (SD).

Clenbuterol yielded an increase in the BR (mean = 12.4 ± 0.2 U). We performed the epitope analyses employing specific competitive inhibitory peptides of the second and third extracellular loops of the β_2_-AR (Figure 3). Loop III but not loop II peptides neutralized the inhibitory β_2_-AAbs. These data indicate that inhibitory β_2_-AAbs in the sera of patients with PEXS are directed against the third extracellular loop of the β_2_-AR.

### The Inhibitory β_2_-AAbs Are of IgG3 Subclass

**FIGURE 4:**
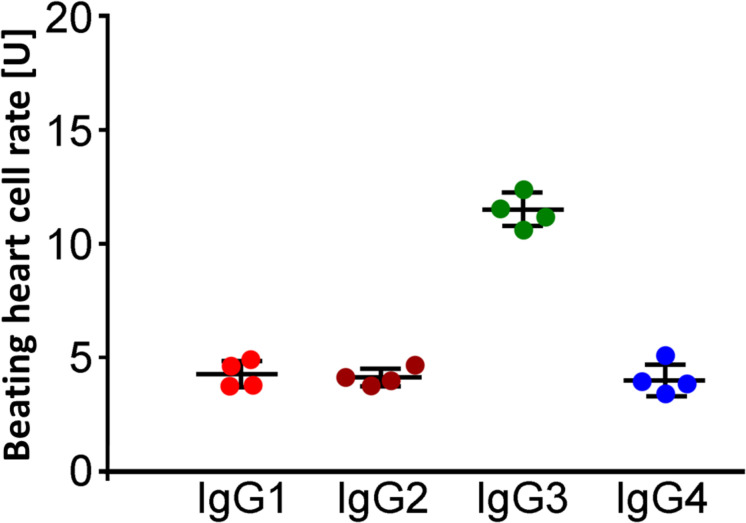
Identification of the IgG3 subclass of the inhibitory anti–β_2_-AAbs in patients with PEX syndrome (PEXS). IgG subclass analysis was done by IgG, prepared from sera of patients with PEXS by direct ammonium sulfate precipitation. The samples were treated with murine monoclonal anti-human IgG1, IgG2, IgG3, and IgG4 antibodies prior to application to the cardiomyocyte bioassay. Beating heart cell rates after immune precipitation of IgG1 (red), IgG2 (dark orange), IgG3 (green), and IgG4 (blue) in the presence of additional application of clenbuterol (Cb); absolute date, mean ± standard deviation (SD); 1 unit (U) reflects 4 beats/min. Only immune precipitation of IgG3 removed the inhibitory effect of inhibitory anti–β_2_-AAbs on the Cb effect. Consequently, the inhibitory anti–β_2_-AAbs are of IgG3 subtype.

The analyses of the IgG subtype were performed by immune precipitation with antibodies against the immunoglobulins IgG1–4. The residual inhibitory β_2_-AAb activities after preincubation with IgG subclass–specific antibodies were 4.3 ± 0.3 U (IgG1), 4.1 ± 0.2 U (IgG2), 11.7 ± 0.4 U (IgG3), and 4.0 ± 0.3 U (IgG4), respectively. The activity of the inhibitory β_2_-AAbs was eliminated by preincubation with anti-IgG3 antibodies. Thus, the inhibitory β_2_-AAbs are of the IgG3 subclass (Figure 4).

### Sera of Patients With PEXG Contain Agonistic, Yet No Inhibitory β_2_-AAbs

**FIGURE 5:**
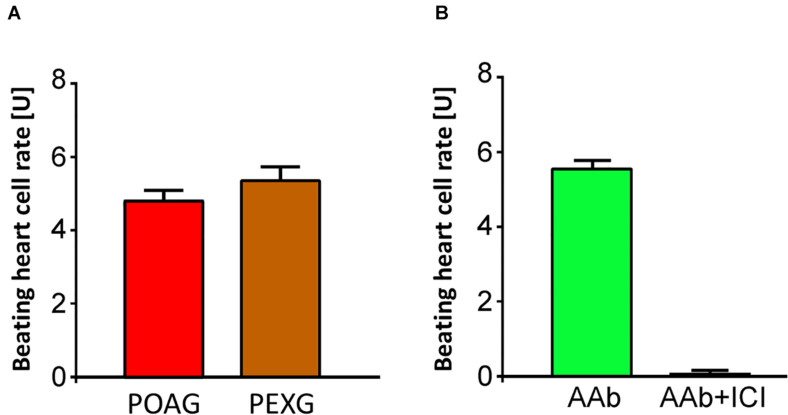
Agonistic β_2_-agAAbs in sera of open-angle glaucoma. **(A)** Beating heart cell rates after incubation with sera of patients with primary open-angle glaucoma (POAG, red) and PEX glaucoma (PEXG, orange); mean ± standard deviation (SD); 1 unit (U) reflects 4 beats/min. An increase of the beating heart cell rate was observed in POAG and PEXG, showing its agonistic effect. Beating heart cell rate of POAG and PEXG did not differ significantly (*p* > 0.05). **(B)** Beating heart cell rates after application of sera without (green) and with ICI-118,551 (black): the agonistic effect of the β_2_-AAbs is shown. The agonistic nature of β_2_-agAAbs activated the receptor with consequent increase of the beating heart cell rate. These agonistic β_2_-agAAbs were observed in sera of patients with open-angle glaucoma.

**FIGURE 6:**
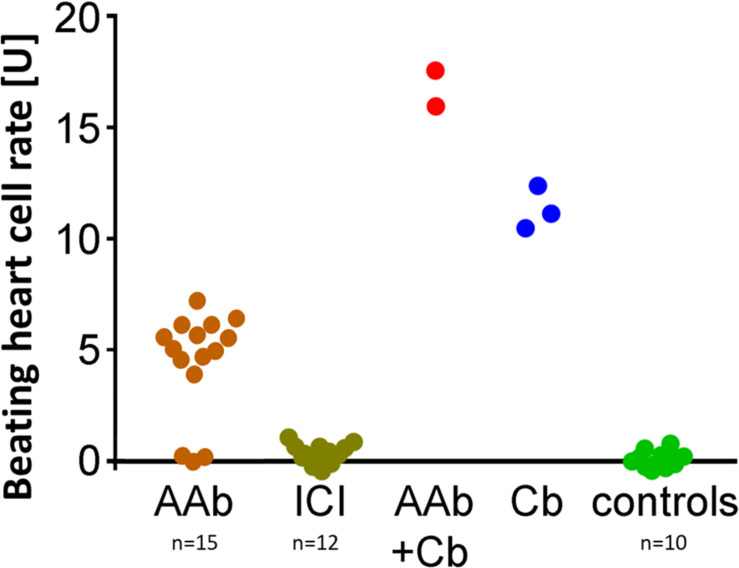
Synergistic β_2_-AAbs enhanced the effect of Cb alone and enhanced the agonistic effect of β_2_-agAAbs. Beating cell rates after incubation of live rat cardiomyocyte cells with patient sera from PEX glaucoma (PEXG): sera without (orange) and with the β_2_-blocker ICI-118,551 (gray–green); synergistic β_2_-AAbs (red) after coincubation of the beating heart cells with clenbuterol (Cb); reference: beating heart cell rate after incubation with clenbuterol alone (blue) and of control subjects (green). One unit (U) reflects 4 beats/min. Sera of PEX glaucoma showed synergistic and agonistic β_2_-AAbs: agonistic β_2_-AAbs were present as beating heart cell rates were increased compared to controls, reaching Cb effect alone. Synergistic β_2_-AAbs enhanced the effect of Cb alone and enhanced the agonistic effect of β_2_-agAAbs. ICI blocked the effect of the β_2_-adrenergic receptor AAbs demonstrating the specificity for the β_2_-adrenergic receptor on the beating heart cells.

In contrast to patients with PEXS and controls, 12 (80%) of 15 of those with PEXG had β_2_-agAAbs. The mean was 4.55 ± 0.6 U in all the PEXG patients (Figure 5). The BR between patients with PEXG and those with POAG did not differ (3.9 ± 0.7 U; *p* > 0.05). In contrast, no inhibitory β_2_-AAbs were to be observed. Figure 6 shows that 2 (13.3%) of the 15 of the patients with PEXG showed synergistic β_2_-AAbs that markedly increased BR values after coincubation with 1 μM clenbuterol (16.3 ± 0.6 U vs. 11.5 ± 0.1 U for clenbuterol alone). Only 1 (6.7%) of 15 PEXG patients showed neither agonistic nor inhibitory β_2_-AAbs.

**FIGURE 7:**
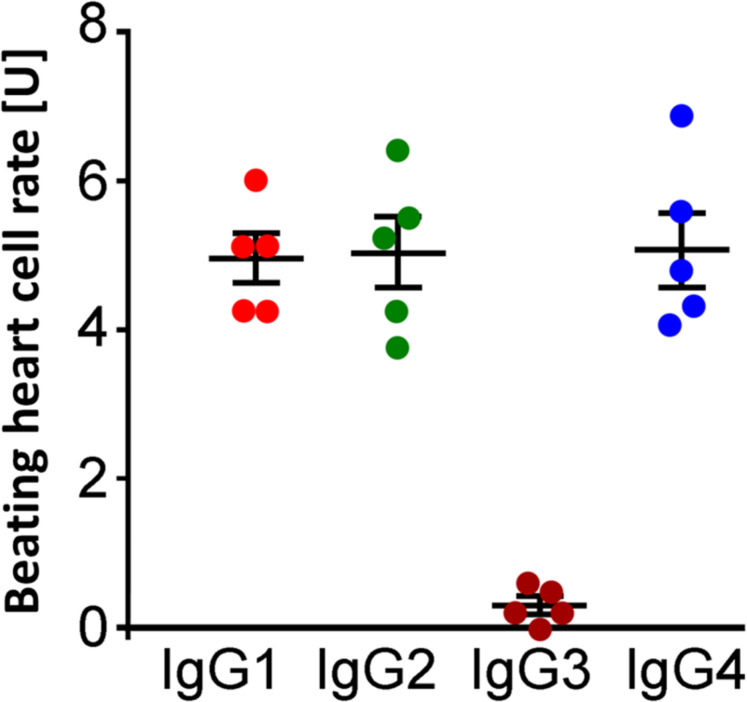
Identification of IgG3 subclass of β_2_-agAAbs from patients with PEX glaucoma. Beating heart cell rates after incubation pretreated with anti-human IgG1 (red), IgG2 (green), IgG3 (dark orange), and IgG4 (blue); absolute date, mean ± standard deviation (SD); 1 unit (U) reflects 4 beats/min. The increase in the beating heart cell rate was eliminated only after incubation with anti-human IgG3 (dark orange). Only peptides against IgG3 inhibited the agonistic nature of β_2_-agAAbs, visualized by the increase of the beating heart cell rate. Consequently, the agonistic β_2_-agAAbs are of the IgG3 subtype.

Analysis of IgG subclasses showed that the β_2_-agAAbs were of the IgG3 subclass (Figure 7), as only precipitation with IgG3 antibodies eliminated the increase in the BR value (0.29 ± 0.1 U). Consistently, no alterations of the BR were seen after precipitation of IgG1 (5.0 ± 0.3 U), IgG2 (5.0 ± 0.5 U), and IgG4 (5.1 ± 0.5 U). Epitope analyses of β_2_-AR showed that the β_2_-agAAbs were directed against the second extracellular loop of this G-protein–coupled receptor (in full) as described previously (Junemann et al., 2018).

## Discussion

PEXS and PEXG are age-related ocular and systemic diseases associated with the accumulation of abnormal fibrillar material in intraocular and extraocular tissues (Elhawy et al., 2012; Scharfenberg and Schlotzer-Schrehardt, 2012; Schlotzer-Schrehardt and Khor, 2020). With an annual incidence of 1.81%, PEXS is a common condition (Astrom et al., 2007), yet its prevalence varies worldwide (Cashwell and Shields, 1988; Krishnadas et al., 2003; Topouzis et al., 2019). It accounts for 25% and up to 70% of all open-angle glaucoma (OAG), depending on the country (Ritch, 1994; Mitchell et al., 1999; Andrikopoulos et al., 2009). Although PEXS is a major risk factor for glaucoma, it is important to note that only 30–40 of all individuals with PEXS will develop PEXG (Thorleifsson et al., 2007). In longitudinal population-based studies, the probability to develop glaucoma after 10–12 years was found to be 9–15% (Jeng et al., 2007; Topouzis et al., 2019). PEXG is a hypertensive type of glaucoma associated with high levels and fluctuations of the IOP, as well as an increased outflow resistance (Kozobolis et al., 2012; Huchzermeyer et al., 2015). Eyes of patients with PEXS tend to have higher IOP levels as healthy eyes but can be normotensive as well (Koz et al., 2009). Although PEXS is an established risk factor for PEXG (Mitchell et al., 1999; Topouzis et al., 2011), the exact relationship between the two conditions is not well understood (Topouzis et al., 2019). Similarly, it remains unclear why only a proportion of PEXS patients develop glaucoma, whereas the majority of them will not (Anastasopoulos et al., 2015; Schlotzer-Schrehardt and Khor, 2021). In a longitudinal population-based study, statistically significant risk factors for the development of PEXG among PEXS patients included higher levels of IOP, with 26% increased risk per mm Hg, and a history of heart attack (Topouzis et al., 2019). It is also conceivable that an underlying defect in the flow dynamics of the aqueous humor or additional involvement of glaucoma susceptibility genes may predispose to glaucoma development in PEXS eyes (Schlotzer-Schrehardt and Naumann, 1995; Kozobolis et al., 2012; Founti et al., 2015; Huchzermeyer et al., 2015; Schlotzer-Schrehardt and Khor, 2021). It has been suggested that in addition to IOP, further factors contribute to the development of glaucoma in patients with PEXS. These include genetic, epigenetic, environmental, and tissue factors such as increased susceptibility of the lamina cribrosa to optic nerve damage (Topouzis et al., 2011; Braunsmann et al., 2012; Hohberger et al., 2018; Schlotzer-Schrehardt and Khor, 2021). Autoimmune mechanisms have also been suggested to participate. Interestingly, there was no difference between the antibody patterns in the aqueous humor of patients with POAG and PEXG. This indicates common pathomechanisms in dysregulation of aqueous humor dynamics and IOP in these glaucoma subtypes (Joachim et al., 2007). Previously, we have identified specific agAAbs affecting the β_2_-AR in sera of patients with PEXG, as has been also shown for patients with POAG (Hohberger et al., 2019a). A subsequent study showed that β_2_-agAAbs were also present in aqueous humor samples of patients with POAG or PEXG (Hohberger et al., 2020). It has been hypothesized that via an impaired blood-aqueous barrier in PEXS/PEXG, serum proteins including β_2_-agAAbs enter the anterior chamber, as observed in a previous study (Schlotzer-Schrehardt and Naumann, 2006). Alternatively, the β_2_-agAAbs may be produced locally in ocular tissues. As the β_2_-agAAbs already occur in very early stages of glaucoma and OHT [e.g., patients without optic disc alterations, yet increased IOP (OHT)] (Junemann et al., 2018), we were interested to find out if patients with risk of PEXG (e.g., PEXS) harbor β_2_-agAAbs. The findings of the present study showed that patients with PEXS lacked any agAAbs. Instead, 80% contained inhibitory β_2_-AAbs of the IgG3 subtype, which is directed against the third extracellular loop of the β_2_-AR. In contrast, patients with PEXG had β_2_-agAAbs, yet no inhibitory ones. Interestingly, synergistic β_2_-agAAbs were to be observed in the sera of two patients with PEXG, overtopping the effect of the β_2_-adrenergic agonist clenbuterol. The presence of synergistic β_2_-AAbs had never been published before. The data of the present study suggest that inhibitory AAbs present in the majority of PEXS patients may contribute to maintenance of normal IOP by reducing aqueous humor production and counterbalance the age-related decline in outflow facility. In addition, we hypothesized that agAAbs may contribute to an increase in IOP by stimulating aqueous humor production in the presence of increased outflow resistance due to abnormal deposits in the trabecular meshwork. This scenario potentially contributes to the etiopathogenesis of glaucoma.

Immune and autoimmune factors were recently reported to be involved in the pathogenesis of OAG (Tezel et al., 2007; Tezel and Fourth ARVO/Pfizer Ophthalmics Research Institute Conference Working Group, 2009; Joachim et al., 2013; Rieck, 2013; Kamat et al., 2016; Von Thun Und Hohenstein-Blaul et al., 2016; Chen et al., 2018; Hohberger et al., 2019a; Tsai et al., 2019). A spectrum of various autoantibodies, mostly directed against retinal antigens, has previously been detected in the sera of patients with PEXS and PEXG (Dervan et al., 2010; Altintas et al., 2012; Huflejt et al., 2014; Mazeikaite et al., 2016) and in the aqueous humor of patients with PEXG (Joachim et al., 2007). In the latter study, there were no significant differences in these antibody patterns between PEXG and POAG. β_2_-AgAAbs levels of PEXG and POAG were similar as well. The present study showed for the first time inhibitory β_2_-AAbs in the sera of patients with PEXS. Inhibitory β_2_-AAbs block the function of the β_2_-AR. This finding is supported by the clinical feature of a less pronounced mydriasis after application of 10% phenylephrine in PEXS eyes compared to eyes without PEXS (Puska, 2002). As reported in a previous study, the local level of β_2_-agAAbs in the aqueous humor was increased compared to the systemic levels (Hohberger et al., 2020). Thus, we propose that adrenergic β_2_-AAbs can be locally produced in ocular tissues. The adrenergic agAAbs in aqueous humor are the basis of our hypothesis of inhibitory β_2_-AAbs in PEXS: due to blocking of β_2_-AR by inhibitory β_2_-AAbs, endogenous catecholamine will shift to alternative AR. Consecutively, AR receptors will be uncoupled (acute response), phosphorylated, and internalized (desensitization of AR) (see Wallukat, 2002). Application of phenylephrine induces mydriasis by contraction of the iris dilatator in unaffected eyes. This mydriatic effect might be reduced in cells of the iris dilatator with desensitized AR.

The exact etiopathogeneses of PEXS and PEXG are still under debate. Two main questions remain unanswered: what is the pathomechanism behind the unilateral clinical manifestation of PEXS/PEXG? And why does only a subgroup of the eyes with PEXS convert to PEXG? It is assumed that the basis of PEXS is a genetic predisposition (e.g., LOXL1). In order to present the clinical features with PEX material on ocular structures, several endogenous and/or exogenous factors are engaged in this complex interplay. Inhibitory, agonistic, and even synergistic β_2_-AAbs turned out to be a common feature in PEXS/PEXG patients in samples of the present cohort (80%); thus, we propose that this form of autoimmunity contributes to the clinical characteristics of PEXS/PEXG. Patients with PEXS show inflammatory markers in their sera (Gonzalez-Iglesias et al., 2014) and aqueous humors [e.g., interleukin (IL-6), IL-8] (Koliakos et al., 2001; Zenkel et al., 2010; Borras, 2018), arguing for an underlying subclinical inflammation. According to the findings of the present study, we propose that because of this inflammatory status, autoimmune mechanisms occur with the generation of autoantibodies, being inhibitory (PEXS), agonistic, or synergistic (PEXG). The specific adrenergic agAAbs might further increase the variation of each clinical entity. Inhibition or overstimulation of the AR might result in a dysregulation of the homeostatic adrenergic balance. This non-canonical receptor–immune complex interaction inhibits or overactivates the adenylate cyclase, causes receptor internalization (due to overstimulation by β_2_-agAAbs), or even enhances AR expression in the sarcolemma membrane (e.g., due to inhibition by inhibitory β_2_-AAbs) (Wallukat et al., 2002). If this molecular dysbalance has sufficient “power,” clinical features might occur in patients with PEXS/PEXG (imbalanced autonomic theory; see Szentiványi, 1968).

Inhibitory β_2_-AAbs were observed in sera of patients with allergic bronchial asthma for the first time (Wallukat and Wollenberger, 1991). The present study showed inhibitory β_2_-AAbs in patients with PEXS as second clinical entity for the first time. Inhibitory β_2_-AAbs bind to β_2_-AR in a competitive way (Wallukat and Wollenberger, 1991). Thus, their binding site to the third loop seems to be different from the β_2_-agAAbs that bound to the first or second extracellular loop of the receptors. After binding, the AR function will be blocked, reducing the activity of adenylate cyclase with consecutive enzymatic dysbalance (Wallukat, 2002; Johnson, 2006). On the contrary, agonistic and even synergistic β_2_-AAbs were present in patients with PEXG. As described in previous studies, we propose that β_2_-agAAbs contribute to the increased IOP in patients with OAG (Junemann et al., 2018; Hohberger et al., 2019a). The presence of synergistic β_2_-AAbs in sera of patients with PEXG argues for the distinct IOP pattern in these eyes. IOP in PEXG is elevated as in all OAG eyes, yet with strongly enhanced diurnal fluctuations and worsened disease progression compared to POAG. This theory might be parallel to the “β-adrenergic theory of the atopic abnormality in bronchial asthma” (Szentiványi, 1968). Szentiványi (1968) hypothesized that decreased β-adrenergic sensitivity results in an autonomic dysbalance, featuring the typical clinical characteristics of asthma. Szentivanyi postulated that “its ubiquitous character suggests, therefore, that the ultimate clinical manifestation of the fundamentally same atopic abnormality will be determined by the type of cell primarily involved, that is, by the specialized cell system of the multicellular organism which harbors primarily the postulated enzymatic abnormality.” It would be of interest, if these autoimmune phenomena will change during disease progression: if either one agAAb subtype will disappear and another one appears, or the presence of a specific adrenergic agAAb subtype would potentially influence the clinical characteristics: PEXS (inhibitory β_2_-AAbs) or entities with increased IOP [OAG including PEXG and POAG (Hohberger et al., 2019a, 2020) and OHT (Junemann et al., 2018); β_2_-agAAbs, Table 2]. A longitudinal follow-up study would be of interest to focus on the pattern of adrenergic AAbs in progressive glaucoma patients. This offers the potential function of β_2_-AAbs as novel biomarkers, supporting diagnosis and monitoring of PEXS, PEXG, and POAG. It potentially provides novel therapeutic targets for one of the most serious types of glaucoma (PEXG), as well as the most common glaucoma form (POAG). The data of the present study already enable the use of adrenergic autoantibodies as novel biomarker, differentiating between PEXS and PEXG. Analysis of a potential presence of adrenergic AAbs locally in aqueous humor of PEXS and PEXG would give additional novel hints insight toward locus of production and pathogenesis.

**TABLE 2:** Autoantibodies targeting the adrenergic β_2_-AR in glaucoma suspects and patients with glaucoma: pseudoexfoliation syndrome (PEXS), pseudoexfoliation glaucoma (PEXG), ocular hypertension (OHT), primary open-angle glaucoma (POAG), secondary open-angle glaucoma (SOAG), and pigment dispersion (PD).

	Clinical characteristics			Serum	Aqueous humor	References
	Increased IOP	Glaucomatous visual field defect	Glaucomatous optic disc			
PEXS	−	−	−	Inhibitory β_2_-AAbs		Present study
PEXG	+	+	+	Agonistic β_2_-AAbs		Hohberger et al., 2019a
					Agonistic β_2_-AAbs	Hohberger et al., 2020
				Synergistic β*_2_-AAbs*		Present study
OHT	+	−	−	Agonistic β_2_-AAbs		Junemann et al., 2018; Hohberger et al., 2019a
Preperimetric POAG	+	−	+	Agonistic β_2_-AAbs		Hohberger et al., 2019a
POAG	+	+	+	Agonistic β_2_-AAbs		Junemann et al., 2018
					Agonistic β_2_-AAbs	Hohberger et al., 2020
SOAG due to PD	+	+	+	Agonistic β_2_-AAbs		Hohberger et al., 2019a

## Conclusion

The presence of inhibitory, agonistic, and even synergistic β_2_-AAbs in sera of PEXS and PEXG, respectively, argues for an imbalanced adrenergic control of the IOP (imbalanced autonomic theory). Each adrenergic agAAb subtype might contribute to the typical clinical characteristics (PEXS, PEXG). Whereas patients with PEXS showed inhibitory β_2_-AAbs, none of these AAbs were to be observed in the sera of PEXG, yielding agonistic and synergistic β_2_-AAbs. These observations argue for an involvement of the β_2_-AAbs early in the pathogenesis of glaucoma. In addition, β_2_-AAbs might offer a novel option as biomarker or therapeutic target in glaucoma.

## Data Availability Statement

The original contributions presented in the study are included in the article/supplementary material, further inquiries can be directed to the corresponding author/s.

## Ethics Statement

The studies involving human participants were reviewed and approved by the Ethics Committee of the University of Erlangen-Nuremberg. The patients/participants provided their written informed consent to participate in this study.

## Author Contributions

US-S, RK, MH, and GW conceived the idea. GW performed the experimental work. BH wrote the draft of the manuscript. CM interpreted the results and revised the manuscript. RL revised the manuscript. BH, MH, and LM performed statistical analysis. All authors contributed to the article and approved the submitted version.

## Conflict of Interest

RK as the patent EP1832600A1. MH has the patent EP1832600A1. GW was employed by Berlin Cures GmbH, Berlin, Germany and holds the patent P1832600A1. The remaining authors declare that the research was conducted in the absence of any commercial or financial relationships that could be construed as a potential conflict of interest.

## Publisher’s Note

All claims expressed in this article are solely those of the authors and do not necessarily represent those of their affiliated organizations, or those of the publisher, the editors and the reviewers. Any product that may be evaluated in this article, or claim that may be made by its manufacturer, is not guaranteed or endorsed by the publisher.
